# A Note on the Definition and the Development of Cerebellar Purkinje Cell Zones

**DOI:** 10.1007/s12311-012-0367-5

**Published:** 2012-03-07

**Authors:** J. Voogd

**Affiliations:** Dept. of Neuroscience, Erasmus Medical Center, Rotterdam, The Netherlands

**Keywords:** Purkinje cell zones, Climbing fibers, Purkinje cell birthdates, Cerebellum development

## Abstract

The definition of Purkinje cell zones by their white matter comprtments, their physiological properties, and their molecular identity and the birthdate of their Purkinje cells will be reviewed.

The cerebellar Purkinje cell layer generally is described as a homogeneous structure. Its subdivision into discrete longitudinal zones with abrupt changes in their connectivity at their borders was based on the observation of the subdivision of the cerebellar white matter of the cerebellum of the cat and the ferret into compartments. In transverse sections a regular pattern of mediolaterally disposed bundles of medium-sized myelinated Purkinje cell axons was observed, separated by darker staining slits consisting of smaller fibers (Fig. 1) that was repeated in each successive folium. When these borders were traced in serial sections, they merge with these borders between the cerebellar nuclei. Each compartment, therefore, contains a particular cerebellar nucleus. It was concluded that each of the white matter compartments channels Purkinje cell axons from a Purkinje cell zone to a particular cerebellar nucleus, indicated with arrows in Fig. 1. Six compartments and corresponding Purkinje cell zones A, B, C1–3, and D can be distinguished. In the crus II of the ansiform lobule, the paramedian lobule, and the paraflocculus, the D compartment is divided into D1 and D2. Experiments with discrete lesions or injections of antegrade axonal tracers in subnuclei of the inferior olive of the cat resulted in the degeneration of bundles of olivocerebellar fibers that were located within particular white matter compartments and terminated on the Purkinje cells of the corresponding zone and its target nucleus (indicated with arrows in Fig. 1). The corticonuclear and the olivocerebellar projection, therefore,are congruent [1–3].

**Fig. 1:**
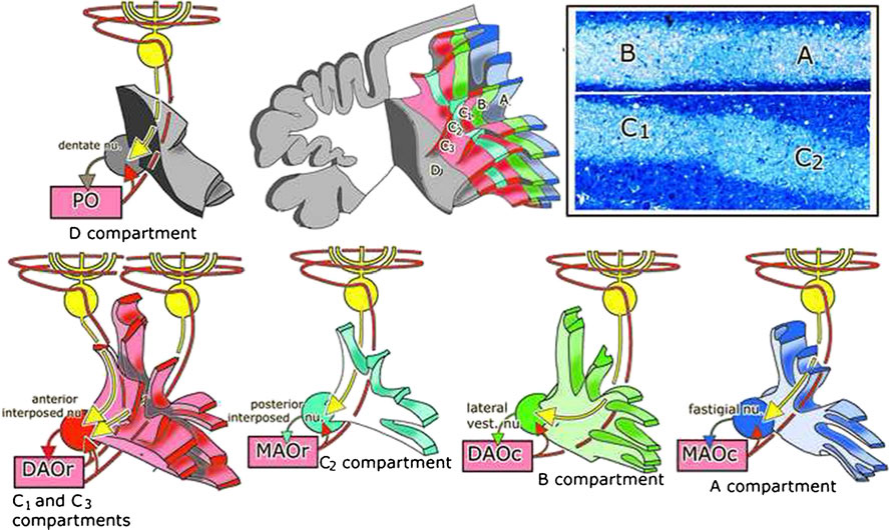
White matter compartments and Purkinje cell zones of the ferret. *A* Reconstructions of the white matter compartments of the anterior lobe. Individual compartments channel Purkinje cell axons (*yellow arrows*) of the corresponding Purkinje cell zone to a particular cerebellar nucleus and the climbing fibers from a subnucleus of the inferior olive that innervate this Purkinje cell zone and its target nucleus (*red arrows*). *Inset* white matter compartments contain bundles of medium-sized myelinated Purkinje cell axons, embedded in smaller fibers. At the border of the compartments *A* and *B* (*upper panel*) and *C*_*1*_ and *C*_*2*_ (*lower panel*) only small fibers are present. *A–D* Purkinje cell zones A–D, *C* subnucleus C of the MAOc, *DAOc/r* caudal/rostral dorsal accessory olive, *DC* dorsal cap, *DM* dorsomedial subnucleus, *int* intermediate MAO, *MAOc* caudal medial accessory olive, *MAOc/r* caudal/rostral medial accessory olive, *PO* principal olive

In the same period, Olav Oscarsson and his collaborators from the Department of Physiology in Lund, Norway recorded positive surface-climbing fiber potentials and Purkinje cell complex spikes from the anterior lobe of the cat in preparations where they had transected the spinal cord, except for one of the funiculi. Oscarsson found that climbing fiber potentials on stimulation of peripheral nerves always were located in parasagittal zones [4]. Oscarsson’s students Ekerot and Larson [5, 6], who studied the dorsal funiculus spino-olivocerebellar climbing fiber path, recognized the A, B, C1–3, and D zones and added three new zones that had not been identified in the anatomical studies. The X and CX zones, located between A and B and lateral to C1, respectively, receive their climbing fibers from intermediate regions of the medial accessory olive. In the lateral anterior lobe, they identified the D2 or Y zone transversely branching olivocerebellar fibers from the rostral dorsal accessory olive, terminated as climbing fibers in pairs of noncontiguous zones: D2 and the lateral C3 zone, or medial C3 and D1. Apparently these zones belong to the same system, receiving peripheral input from the rostral dorsal accessory olive through climbing fibers branching between its components

More recently Voogd et al. [7] and Sugihara and Shinoda [8] mapped the olivocerebellar projection in rats, and compared it to the pattern of alternating aldolase C (zebrin II)-positive or negative bands [9] (Fig. 2, left panel). Ekerot’s X and Y zones could be identified in the rat, Y corresponding to Buisseret’s D0 zone [10]. In addition their rodent-specific A2 zone could be identified (Fig. 2, right panel). Climbing fiber zones were found to be congruent with the zebrin pattern. C2, D1, and D2 zones are zebrin positive, X, B, C1, C3, and D0 zebrin negative. A and A2 consist of a multitude of narrow, alternating zebrin-positive and -negative bands.

**Fig. 2:**
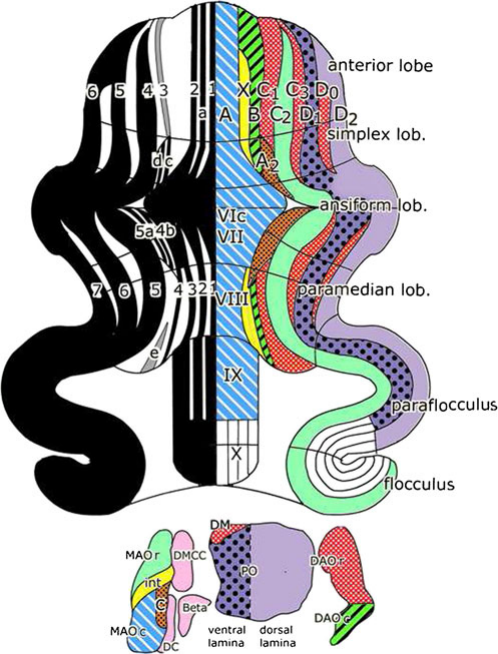
Comparison of maps of the flattened cerebellar cortex showing the zebrin II (*left panel*) band pattern with the olivocerebellar projection zones *A*–*D* (*right panel*). For abbreviations see Fig. 1

Pukinje cell zones develop early from a series of superficial, mediolaterally disposed Purkinje cell clusters [11]. Recently Hashimoto established in mice that Purkinje cells of different adult zones are born on different dates, using adenoviral vectors injected into the midbrain ventricle of mouse embryos on different embryonic days [12]. When different cohorts of labeled Purkinje cells were mapped in sections counterstained for aldolase C (zebrin II), the birthdates of the Purkinje cells of the identified adult zones could be established [13] (Fig. 3). It can be concluded that Purkinje cells of the rostral MAO-innervated X and C2 zones and of D0 are born early at E10.5. Purkinje cells of D1 and D2 are born on E10.5 and 11.5. C1 and C3 Purkinje cells are a late mixture born on E11.5 and 12.5. Apparently, the molecular identity of the Purkinje cells is not established at their birth. Cohorts born on E10.5 develop into both zebrin-positive (C2) and zebrin-negative (X, D0) bands. As an analogue to the late birth of C1 and C3 Purkinje cells in mice, Kappel [14] noticed in macaque fetuses that the corresponding Purkinje cell clusters migrate to the cerebellar surface much later where they penetrate between the early arriving clusters of the A, C2, and D zones (Fig. 4).

**Fig. 3:**
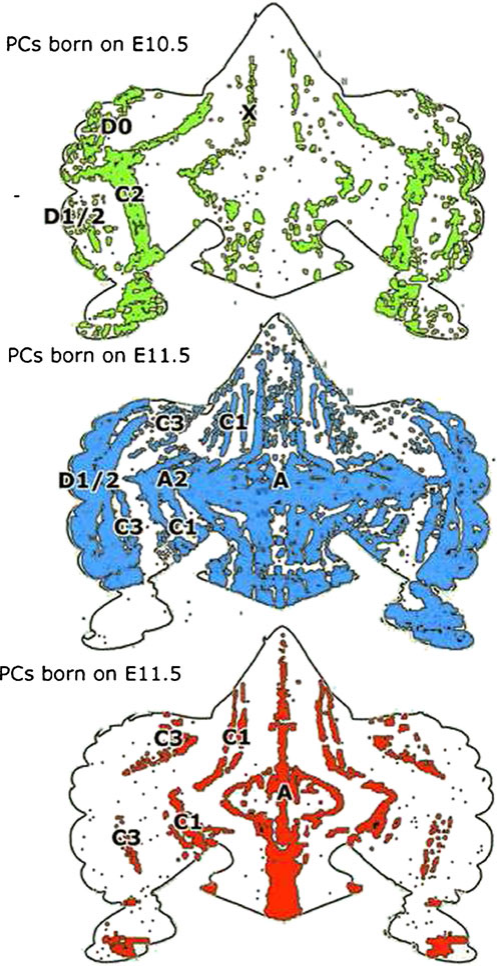
Distribution and zonal identification of Purkinje cells born on E10.5 (*B*, *green dots*), PCs born on E11.5 (*C*, *blue dots*), and PCs born on E12.5 (*D*, *red dots*) plotted on a flattened map of the cerebellar cortex of the mouse. Modified from Namba et al. [13]

**Fig. 4:**
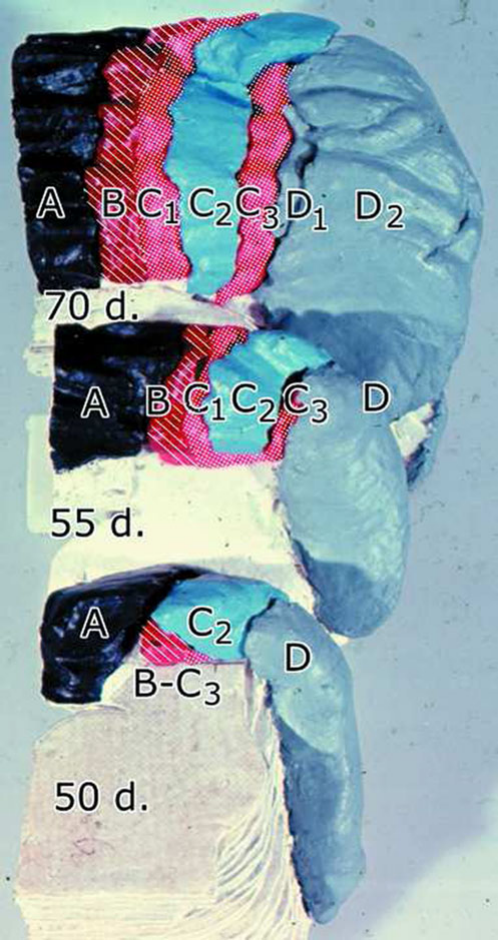
Anterior aspects of styrofoam reconstructions of the cerebellar anlage of three 50-, 55-, and 70-day rhesus monkey fetuses, showing the emergence of the *B*, *C*_*1*_, and *C*_*3*_ Purkinje cell clusters between the early arriving *A*, *C*_*2*_, and *D* clusters. From Kappel [14]
